# Human prostate supports more efficient replication of HIV-1 R5 than X4 strains ex vivo

**DOI:** 10.1186/1742-4690-5-119

**Published:** 2008-12-31

**Authors:** Anna Le Tortorec, Anne-Pascale Satie, Hélène Denis, Nathalie Rioux-Leclercq, Laurence Havard, Annick Ruffault, Bernard Jégou, Nathalie Dejucq-Rainsford

**Affiliations:** 1 Inserm, U625, Rennes, France; 2 Univ Rennes I, Campus de Beaulieu, IFR-140, GERHM, Rennes, F-35042, France; 3 Unité de rétrovirologie, CHU Pontchaillou, Rennes, France; 4 Service d'anatomie et cytologie pathologiques, CHU Pontchaillou, Rennes, France; 5 CNRS/UMR6061, IFR140, Faculté de Médecine, Université de Rennes1, France

## Abstract

**Background:**

In order to determine whether human prostate can be productively infected by HIV-1 strains with different tropism, and thus represent a potential source of HIV in semen, an organotypic culture of prostate from men undergoing prostatic adenomectomy for benign prostate hypertrophy (BPH) was developed. The presence of potential HIV target cells in prostate tissues was investigated using immunohistochemistry. The infection of prostate explants following exposures with HIV-1 R5, R5X4 and X4 strains was analyzed through the measure of RT activity in culture supernatants, the quantification of HIV DNA in the explants and the detection of HIV RNA+ cells *in situ*.

**Results:**

The overall prostate characteristics were retained for 2^1/2 ^weeks in culture. Numerous potential HIV-1 target cells were detected in the prostate stroma. Whilst HIV-1 R5_SF162 _strain consistently productively infected prostatic T lymphocytes and macrophages, the prototypic X4_IIIB _strain and a primary R5X4 strain showed less efficient replication in this organ.

**Conclusion:**

The BPH prostate is a site of HIV-1 R5 replication that could contribute virus to semen. A limited spreading of HIV-1 X4 and R5X4 in this organ could participate to the preferential sexual transmission of HIV-1 R5 strains.

## Background

Although semen represents the foremost vector of HIV-1 dissemination worldwide, the origin of the infected leukocytes and free viral particles contaminating the seminal plasma is still unclear. Semen represents a viral compartment distinct from the blood (reviewed in [1]), strongly suggesting the existence of productive sources within the male genital tract. The male reproductive tract could constitute a sanctuary for HIV-1 replication, since HIV-1 can persist in the semen of seropositive men on highly active antiretroviral therapy (HAART) in the absence of detectable levels of viral RNA in plasma [2-7]. Knowing the sources contributing virus to semen is essential to promoting the design of therapies aimed at eradicating virus from semen.

Semen is composed of secretions and cells from the testes, epididymides, prostate, seminal vesicles and urethral glands. Earlier studies showed that vasectomy has little impact on seminal shedding of HIV-1 [8-11], indicating that testes and epididymides are not the main sources of viral load in semen. The prostate could represent an important source of virus in the semen, since prostate secretions constitute 30% of the seminal fluid and the human prostate is frequently inflammed, encompassing leukocytic infiltrates that may represent target cells for HIV infection (for a review, [12]). We recently demonstrated that the prostate of asymptomatic macaques is productively infected by SIV and displays higher level of infection than the testes and the epididymides [13]. In human, indirect evidence suggests that the prostate is infected and constitutes an early viral reservoir. Thus, prostatic massages performed on asymptomatic HIV-1+ men with no detectable seminal viral load induce the release of virus in the semen [14], and the expressed prostatic secretions from HIV-1+ men with or without ART are consistently positive for HIV RNA [15]. However, for obvious clinical and ethical reasons, in-depth investigations of HIV infection of the prostate of HIV+ asymptomatic men have never been performed.

*In vitro *studies using a number of different human organs, including the testis [16], have proved invaluable for improving the understanding of HIV pathogenesis. To test the hypothesis that the human prostate represents a source of virus in semen, we used an organotypic culture of prostate tissue obtained from men with benign prostate hypertrophy (BPH), a common non-malignant pathology, to assess whether the resident immune cells or other cell types present in this organ are susceptible to infection by HIV-1 strains with various co-receptor requirements.

## Methods

### Chemicals and reagents

The following reagents were used: DMEM, RPMI 1640 (Gibco BRL, Life Technologies, Cergy-Pontoise, France), fetal calf serum (FCS), sodium pyruvate (Eurobio, Courtaboeuf, France), glutamine (Gibco BRL), insulin, vitamin A, vitamin C, vitamin E, dihydrotestosterone, phytohemagglutinin (Sigma, Sigma-Aldrich Corp., St. Quentin Fallavier, France), penicillin-streptomycin (Eurobio) and interleukin-2 (Boehringer-Mannheim, Germany).

### Antibodies, plasmids and cell lines

The following mouse monoclonal antibodies against human proteins and matching isotype controls were used: anti-CD68 (clone KP1), -CD3 (clone F.7.2.38)(7 μg/ml), – HLA-DR (clone TAL.1B5), -Ki-67 (clone MIB-1) (DAKO, Trappes, France), -PSA (clones ER-PR8 and PA05 from Neomarkers), and -CD4 (clone 1F6, Novocastra, Newcastle, UK) with mouse IgG1 (DAKO), anti-CCR5 (clone 45523) and IgG2b (R&D Systems, Minneapolis, MN), anti-CXCR4 (clone 12G5; Dr. J. Hoxie, NIBSC Centralized Facility for AIDS Reagent, UK), – alpha-smooth muscle actin (clone 1A4, DAKO), – p63 (clone 4A4, DAKO) and IgG2a (R&D Systems).

The pNL4.3 plasmid was provided by F. Mamano (Inserm U552, Paris, France). Human T-Lymphoblastoïd C8166 cell line was obtained from the NIBSC Centralized Facility for AIDS Reagent.

### Viral stocks

HIV-1 clade B R5_SF162_, X4_IIIB _strains and R5X4_92US723 _primary isolate (ARP1039.3) were obtained from the NIBSC Centralized Facility for AIDS Reagent and expanded in activated human PBMCs (R5_SF162_, R5X4_92US723_, X4_IIIB_) or in C8166 cells (X4_IIIB_) to provide viral stocks of 40 000 pg/ml for R5_SF162_, 40 000 pg/ml (PBMCs) to 60 000 pg/ml (C8166) for X4_IIIB _and 10 000 pg/ml for R5X4_92US723 _as determined by RT activity assay.

### Organotypic culture of human prostate explants

The protocol was approved by the local ethics committee of Rennes, and informed consent was obtained from the donors. Prostates tissues were obtained at the Rennes University Hospital from healthy, HIV-1 seronegative patients (age range 50–55) who underwent prostatic adenomectomy for BPH. Immediately following surgery, prostate tissues were placed at 4°C in fresh medium and processed within one hour. The benign pathological status was confirmed by histological examination. Prostate tissue from the transition zone and the central zone was dissected into 3-mm^3 ^fragments, and two explants were transferred onto a polyethylene terephtalate (PET) insert in a well of a 12-well tissue culture plate (Falcon Labware; Becton-Dickinson and Co., Lincoln Park, NJ) containing 1 ml of media (DMEM supplemented with antibiotics, 10% FCS, 1 mmol/L sodium pyruvate, 1 mmol/L glutamine, 100 ng/ml vitamin A, 200 ng/ml vitamin E, 50 ng/ml vitamin C, 0.5 μg/ml insulin, and 800 ng/ml 5α-dyhydrotestosterone). Each experimental condition was made up of two wells of two biopsies per well. The culture was established for 17 days in a humidified atmosphere containing 5% CO2 at 37°C, medium was changed every 2 days, and stored frozen at -80°C. Every 2 days, prostate explants were either fixed in neutral buffered 4% formaldehyde or frozen and stored at -80°C.

### Immunohistochemistry and cell count

Immunohistochemistry using avidin-biotin peroxidase complex technique was performed on formaldehyde-fixed, paraffin-embedded tissues as previously described [16]. No staining was ever observed with isotype control antibodies or control serum. Stained positive cells were counted in the total surface of the tissue section in a minimum of three independent cultures using the Cast Grid software (Olympus).

### Infection of prostate explants

Immediately after dissection, two fragments of human prostate tissue (~3 mm^3 ^each) were immersed in either 200 μl (for R5_SF162 _and X4_IIIB_) or 600 μl (for R5X4_92US723_) of cell-free viral supernatant for 3 hours at 37°C and then thoroughly rinsed three times in PBS. The explants were placed in culture as described above, and the culture medium replaced and collected every 2 days throughout the culture and stored at -80°C for RT and viral infectivity assays.

### Reverse Transcriptase (RT) activity assay

Frozen culture supernatants were assayed in duplicates for RT activity using the Lenti-RT activity assay (Cavidi Tech, Uppsala, Sweden) according to the manufacturer's instructions. Unknown values were obtained from the standard curve interpolation and were expressed as pg/ml of RT.

### Simultaneous in situ hybridization and immunohistochemical staining

Identification of cell types expressing HIV-1 RNA was performed by combining radioactive *in situ *hybridization for HIV-1 gag and immunohistochemical staining for cell markers, as previously described [16]. The specificity of the hybridization signal was systematically checked by hybridizing sense probes on parallel sections and anti-sense probes on uninfected prostate tissue.

### Viral infectivity assay

500 μl of prostate culture supernatants collected at day 17 after infection, or 500 μl of viral stock maintained at 37°C for 17 days (used here as a negative control) were ultracentrifuged for 1 hour at 17 000 rpm. Supernatants were discarded and pellets dissolved in 500 μl of RPMI 1640, which was added to 4 × 10^6 ^phytohemagglutinin-activated PBMCs for 3 hours at 37°C. After a 10-minute centrifugation at 1200 rpm, PBMCs were re-suspended in 2 ml of RPMI 1640 supplemented with 5% interleukin-2 and maintained at 37°C for 14 days. Culture medium was changed every 3 days and stored frozen at -80°C for subsequent RT assay.

### Measurement of HIV-1 DNA using TaqMan real-time PCR

Total DNA was extracted using the QIAamp DNA Mini Kit (Qiagen) according to the manufacturer's instructions. Quantitative real time PCR was performed on 250 ng DNA as previously described, using primers and probes for HIV-1 LTR and albumin gene amplification [16,17]. For each donor and each time point, two separate pieces of tissue were analyzed in duplicate. Results were expressed as copy number of Log10 HIV DNA copy number/million cells.

### Statistics

All values are the mean ± SEM. The significance of the differences between values was evaluated using Dunnett test. This test controls the Type I experiment-wise error for comparisons of all samples against a control (described in the figure legends). A value of p < 0.05 was considered statistically significant.

## Results

### Characterization of human prostate in organotypic culture

**Figure 1:**
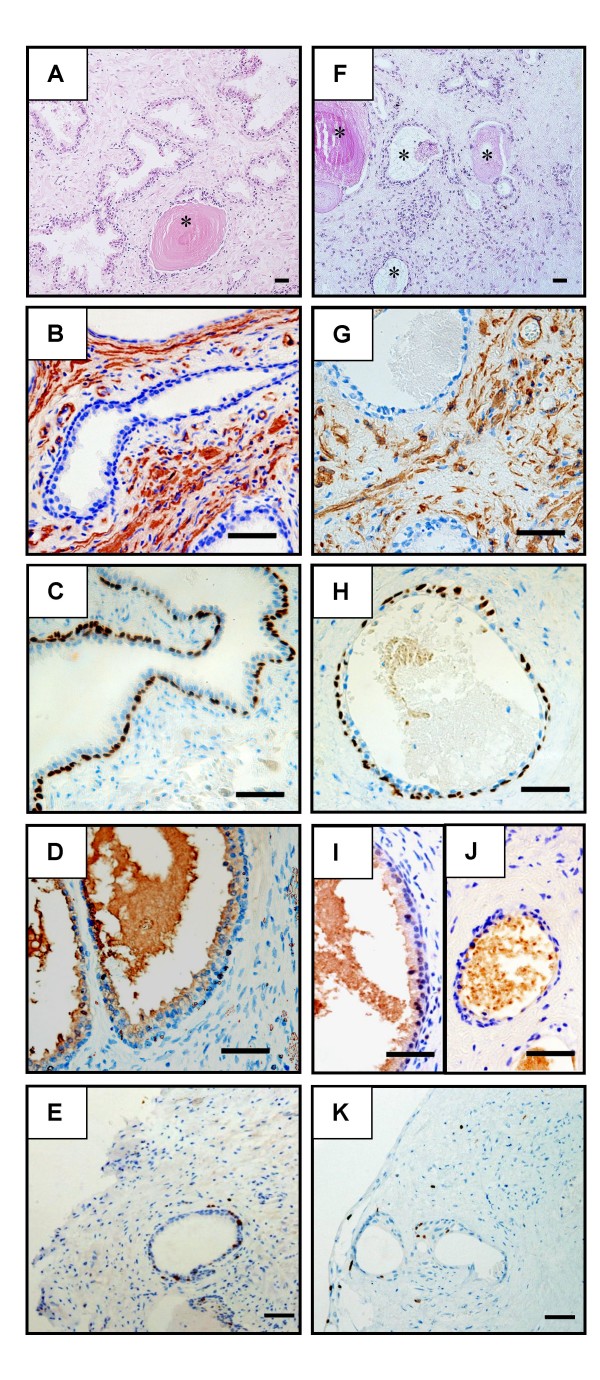
**Characterization of human prostate in organotypic culture**. Paraformaldehyde-fixed paraffin sections were examined morphologically and immunostained for several prostatic cell markers before culture (A-E) and after 4 days (I) or 17 days (F-H, J, K) of culture. (A, F): a mix of normal and hyperplasic glands (identified by stars) were observed in the tissue sections before and after 17 days of culture. The overall morphology of the organ was preserved after 17 days of culture despite a loss of stromal cells in some areas (F). The markers used for the characterization of prostatic cell types were: – α smooth actin for smooth muscle and myofibroblastic stromal cells (B, G); – p63 for basal epithelial cells (C, H); – PSA for secretory luminal epithelial cells (D, I, J); – Ki67 for proliferating cells (E, K). The results are representative of a minimum of three independent cultures performed on prostates collected from different donors. Scale bars = 50 μm.

The morphology and expression of prostatic cell markers were compared in prostate explants before culture and throughout the culture period. Histological examination of prostatic fragments revealed that the tissue architecture was maintained during the 17 day culture period (Fig. 1A versus F). Staining for α smooth actin before culturing revealed the presence of numerous smooth muscle cells and myofibroblasts as well as unstained stromal cells (i.e. fibroblasts, leukocytes and endothelial cells). After 17 days of culture, whilst the same global profile of staining was observed, a diminution of the number of stromal cells, mainly in the centre of the explants was noticed (Fig. 1B versus G). Epithelial basal cells (p63+, PSA-) surrounding the acini were maintained throughout the culture (Fig. 1C, H) and were seen encapsulating the explants from day 4 of culture (data not shown). Luminal columnar epithelial cells (PSA+, p63-) maintained their ability to produce PSA for up to 4 days of culture (Fig. 1D versus I). After 8 days of culture, PSA expression in these cells became undetectable, although PSA positive secretions were still found in acini lumen by day 17 (Fig. 1J). In the explants before and after culture, only a small cell number was proliferating, as shown by the detection of Ki67 (Fig. 1E, K).

### Detection and quantification of potential HIV-1 target cells in human BPH prostate

**Figure 2:**
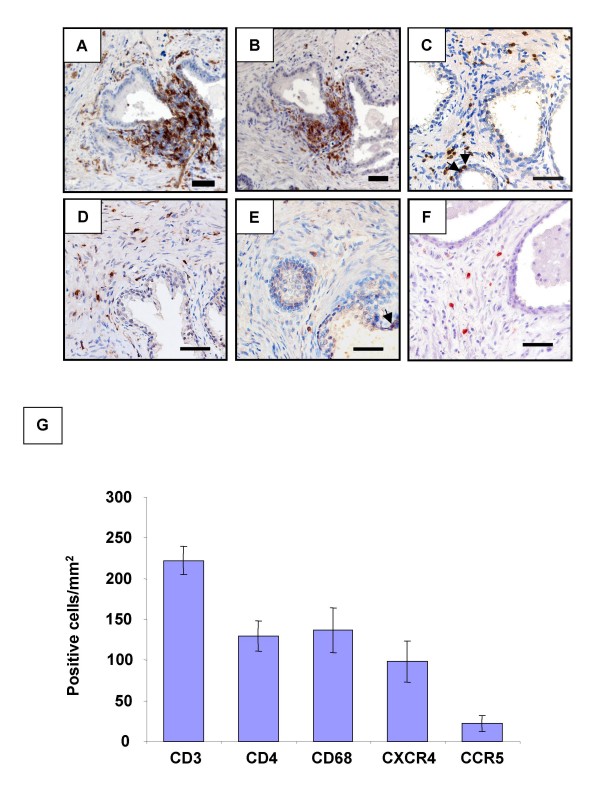
**Presence of potential HIV target cells in the human prostate**. Immunohistochemistry on uninfected prostate sections before culture showed the presence of periglandular foci of HLA-DR+ (A) and CD4+ cells (B, serial section with A) as well as scattered stromal cells staining positive for HLA-DR (A), CD4 (B), CD3 (C), CD68 (D), CCR5 (E) and CXCR4 (F). The arrows point out immune cells inserted within the epithelium-Scale bars = 50 μm; (G): the respective proportions of CD3, CD4, CD68, CXCR4 and CCR5+ cells per surface unit were evaluated on whole prostate sections from a minimum of 3 donors whose explants were subsequently exposed to HIV-1 strains.

Cells staining positive for the activated immune cells marker HLA-DR (Fig. 2A), the T lymphocyte marker CD3 (Fig. 2C), the T helper lymphocyte marker CD4 (Fig. 2B), and the myeloid cell marker CD68 (Fig. 2D) were found within the stroma of fixed prostate tissues from all donors before culture, either scattered or in periglandular foci (mainly composed of HLA-DR+, CD3+ and CD4+cells; serial sections, Fig. 2A, B). These immune cells were also occasionally found as isolated cells inserted within the epithelium of the glands (arrows, Fig. 2). The HIV co-receptors CCR5 (Fig. 2E) and CXCR4 (Fig. 2F) were detected on cells with immune cell type morphology. Quantitative immunohistochemistry indicated that CD3+ T lymphocytes consistently out-numbered CD68+ myeloid cells whilst CXCR4+ cells were more frequently encountered than CCR5+ cells in the prostatic tissue sections examined (Fig. 2G). Staining for all these cell populations was still observed at the end of the culturing period (data not shown).

### Infection of prostate explants with R5, R5X4 and X4 HIV-1 strains

**Figure 3:**
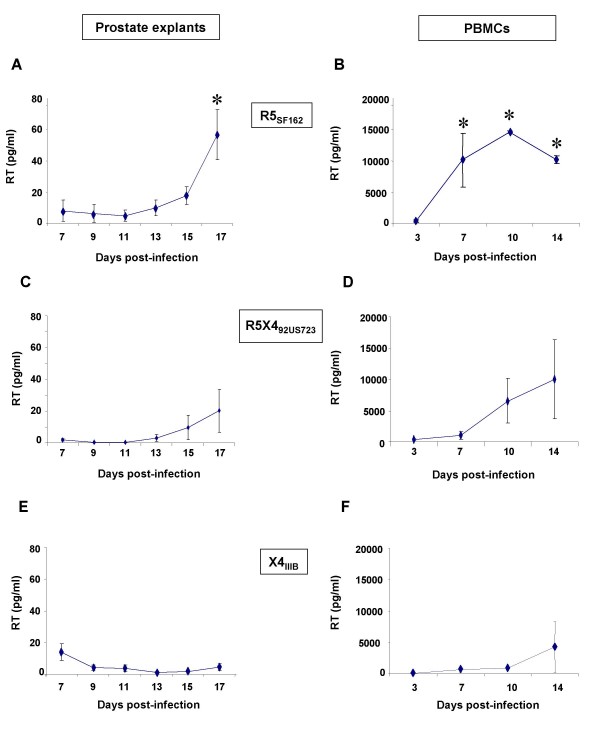
**HIV-1 R5, R5X4 and X4 infection of human prostate in organotypic culture**. RT activity was measured in supernatants of human prostate explants exposed to HIV-1 R5_SF162 _(n = 3 donors) (A), R5X4_92US723 _(n = 3 donors) (C), or X4_IIIB _(n = 5 donors) (E); and in supernatants of activated PBMCs exposed to day 17 supernatants of prostate explant cultures exposed to R5_SF162 _(n = 3) (B), R5X4_92US723_(n = 3) (D) or X4_IIIB _(n = 5) (F). RT activity was never detected in the supernatants of PBMCs infected with an inoculum of the respective viral stocks maintained at 37°C in medium for 17 days. Each dot represents the mean ± SEM of three to five independent cultures (Dunnett test; *, P < 0.05; control: day 7).

Following the incubation of R5_SF162 _with prostate explants from 3 donors, a significant increase in RT activity was consistently observed at day 17 of culturing (Fig. 3A). Viral particles obtained by centrifugation of prostate supernatants collected at the end of the culturing time productively infected activated PBMCs (Fig. 3B), whilst an inoculum of the SF162 viral stock left in the culture medium for 17 days at 37°C did not trigger PBMCs infection, indicating that infection was caused by newly released virions and not by the potential remains of the initial inoculum. Exposure of prostate tissues to R5X4_92US723 _primary isolate induced lower RT activity increases than R5_SF162_, and the RT level was highly variable depending on the patient (Fig. 3C). Accordingly, highly variable levels of productive infection of PBMCs by R5X4-infected prostate culture supernatants were observed (Fig. 3D). In contrast, in supernatants of matched blocks of prostate tissues (same patients) and in prostate tissues from two additional patients exposed to X4_IIIB _grown in C8166, no or extremely low increases in RT activity were detected during the 17 day-culture period (Fig. 3E). The supernatants from X4_IIIB_-exposed prostate cultures triggered either no or very low infection of PBMCs (Fig. 3F). Similar results were obtained using X4_IIIB _stocks grown in PBMCs (data not shown).

**Figure 4:**
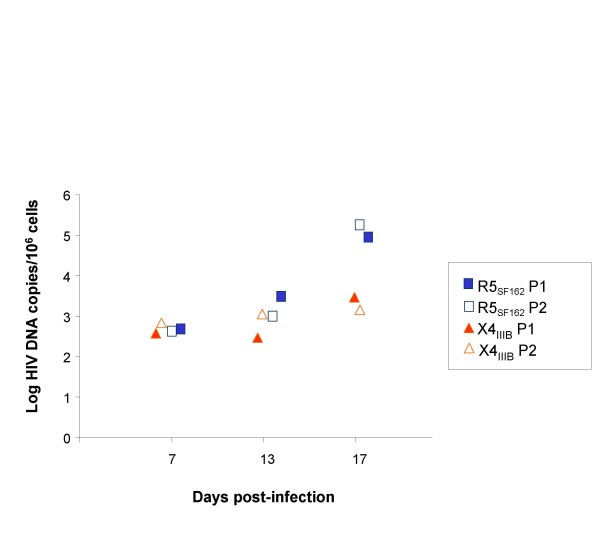
**Accumulation of HIV-1 DNA in prostate explants following exposure to either R5_SF162 _or X4_IIIB_, as assayed for LTR DNA by quantitative real time PCR**. Each dot represents the mean value of 2 paired explants (each tested in duplicate PCR) from one individual. For each virus strain, prostate explants from 2 patients were analyzed.

The quantity of HIV DNA within the prostate explants exposed to either R5_SF162 _or X4_IIIB _was measured using Q-PCR (Fig. 4). HIV-1 DNA level rose from 7 days onwards post-exposure to R5_SF162 _(on average, + ≈ 0.7 Log at day 13 and + ≈ 2.4 Log at day 17), demonstrating productive infection. In contrast, no increase of HIV DNA was observed in prostate explants exposed to X4_IIIB _between day 7 and 13. Although relatively modest (on average ≈ 0.6 Log), an increase was observed at the end of the culturing period (Fig. 4), indicating a low level of X4_IIIB _replication. Of note, the overall morphology and the cellular expression of PSA, p63, α smooth actin and Ki67 of the infected explants at day 4, 11 and 17 of culture was similar to those of uninfected explants (data not shown).

### Localization and characterization of HIV-1 RNA+ cells in BPH prostate explants

**Figure 5:**
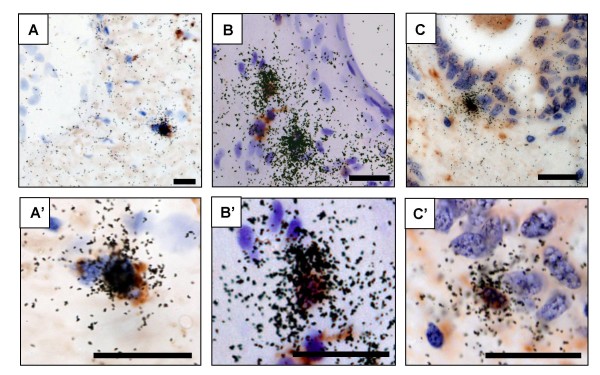
**Localization and characterization of HIV-1 RNA positive cells in the human prostate infected *ex vivo***. Localization of HIV RNA+ cells (black silver grains) in prostate explants exposed for 17 days to R5_SF162 _(A, C) or R5X4_92US723 _(B) using *in situ *hybridization for HIV-1 gag. Combined ISH with immunostaining for cell markers was used to assess co-localization of HIV RNA+ cells with either CD68 (A) or CD3 (B, C). A'-C' correspond to higher magnification of A-C showing cells co-labelled for HIV RNA (black silver grains) and cell marker (brown cells). Scale bars = 20 μm.

Infected cells were localized within the explants by *in situ *hybridization for HIV-1 gag mRNA and characterized by combined immunohistochemistry for cell markers (Fig. 5). In all explants exposed to R5_SF162_, and in those exposed to R5X4_92US723_, mostly isolated and occasional groups of HIV-1 RNA positive cells were detected in the prostatic stroma (Fig. 5A, B). A few HIV+ cells were also observed close to, or inserted within, the prostatic epithelium (Fig. 5C). HIV+ cells co-localized with CD3 (Fig. 5B, B', C, C') and, more rarely, with CD68 (Fig. 5A, A'). Scarcely distributed CD3+HIV-1+ cells were detected in the stroma of X4_IIIB_-exposed prostate explants with low RT activity (data not shown).

## Discussion

Whilst few previous studies have detected HIV-1 within the morphologically abnormal prostate from AIDS deceased men [11,18-20], the susceptibility to HIV-1 infection of the prostate from asymptomatic men has not been thoroughly explored. Here, we used an organotypic culture of prostate tissue from HIV-negative men with benign prostatic hypertophy (BPH) to investigate infection by R5, R5X4 and X4 HIV-1 strains and to determine the nature of the target cells within this organ.

BPH is an extremely common disease (for a review, [21]). Starting from 35 years of age, its frequency increases with age, and it affects 50 to 75% of men over 50 years old. It leads to an enlarged prostate which although benign and non-malignant may necessitate surgery to eliminate discomfort. Most BPH lesions develop in the transition and central zones of the prostate and are composed of epithelial, muscular and/or stromal hyperplasia. Inflammatory infiltrates are often present in and around the glandular or fibromuscular BPH nodules [12]. In our BPH tissues, T lymphocytes (mean of 220 cells/mm^2^), mainly stromal CD4+, were more numerous than CD68+ myeloid cells and were found either scattered or in foci. These results are in agreement with previous qualitative and quantitative studies on immune cell populations within BPH prostates, showing the predominance of T lymphocytes (mean of 195 cells/mm^2^, mainly stromal memory CD4+T cells and a few intraepithelial CD8+ cells) over macrophages and B cells [22-24]. Normal prostate (i.e. from asymptomatic prostate disease-free men) contains scattered stromal and intraepithelial T and B lymphocytes, macrophages and mast cells [23], but also frequently displays focal accumulation of CD4+T lymphocytes in the stroma [25-27]. As a matter of fact, most adult prostate tissues, with or without prostatic pathology, contain various extent of inflammatory infiltrates, most commonly composed of a majority of stromal T cells (reviewed in [12,28]. The source of intraprostatic inflammation is often unknown but might be caused by dietary factors, hormonal exposure variations, infection, or cell injury (for a review [12]).

The culture conditions selected for the organotypic culture of human prostate that we developed from BPH tissues were based both on our own experience of organ culture [29] and on previous descriptions of prostate organ culture selected from the literature [30-32]. The culturing method of using well inserts was chosen for its ease of manipulation in the context of a high security laboratory, as opposed to other methods which required specific material [33]. The culture allowed for the preservation of the overall tissue architecture and the maintenance of all the prostatic cell types for 2.5 weeks ex vivo. Both normal and hyperplasic glands were observed before and after culturing. No increased proliferation of any specific cell type was observed after culturing. The constraint of working on explants of very small size in order to preserve tissue integrity did not allow for accurate quantification of immune cells throughout the culture. However, we unequivocally found that all the immune cell types identified before culturing were still present by day 17.

We demonstrated here for the first time that human prostate from men with BPH is selectively infected by HIV-1 and releases infectious virions. Infected cells were mainly localized within the stroma and were rarely found within the epithelium, whether hyperplasic or normal. Their phenotype corresponded to either lymphocytes or macrophages, which, as previously evidenced by us and others, can be inserted within the epithelium [13,23,25-27]. Epithelial cells in the BPH prostate explants never stained positive for HIV receptors CD4, CXCR4 or CCR5 in immunohistochemistry. This confirms an *in vitro *study showing an absence of CD4, CXCR4 or CCR5 surface expression and the lack of productive infection by either R5 or X4 virus strains of epithelial cells isolated from normal prostates [34]. Thus, unlike the renal epithelium that supports HIV replication *in vivo *[35], the prostatic epithelium does not constitute a site of active HIV infection.

The most efficiently and consistently replicating HIV-1 strain in prostate tissues was the prototypic R5_SF162_. Prostate cultures were also susceptible to infection by a dual tropic primary isolate; albeit replication of the R5X4 virus was much lower than the R5 strain (which could reflect the lower titre of R5X4 viral stock) and was variable depending on the patients. As assessed by RT activity measurement, infected cells were readily detected *in situ *and the viral particles recovered from infected prostate supernatants were able to subsequently infect PBMCs. In contrast, despite a higher titre of viral stock, X4_IIIB _spread in the prostate tissue was somewhat inefficient. This was demonstrated by the absence (or the very low level) of RT activity in infected culture supernatants after 17 days of culture, the scarce detection of infected cells in the tissues, and the fact that supernatants of prostate explants exposed to X4 were rarely able to induce infection of PBMCs. In addition, whilst in the prostate explants infected with R5_SF162 _HIV-1 DNA rose significantly from day 7 onwards, a delayed and much lower increase was observed within the explants exposed to X4_IIIB_. A longer duration of prostate culture may have allowed for a rise in X4 production, as suggested by the increase in X4 DNA towards the end of the culture period; however, since the prostate explant morphologies started to be disrupted after 17 days, the culture was not carried out any further. A similarly favoured replication of R5 over X4 strains has been described in foreskin, cervical, skin epidermal and fetal thymus explants [36-39]. In contrast, whilst rectosigmoid explants supported efficient replication by both R5 and X4 strains, tonsil explants, which displayed low numbers of CCR5+ cells, were less efficiently infected by R5 strains [40]. In foreskin, a predominance of CCR5 over CXCR4 expression by the tissue immune cells was observed, and this could explain the X4 restriction [36]. In our system, CXCR4 was readily detected and, in fact, CXCR4+ cells outnumbered CCR5+ cells. Although differences in antibody sensitivities may have led to an underestimation of CCR5+ cells, the restriction in X4 replication cannot be inferred as emerging from a lack of co-receptor expression. However, we cannot rule out that CXCR4+ cells in the prostate are mainly inactivated/naive CD4+T cells and/or CD8+T lymphocytes, both refractory to productive infection. Another hypothesis is that the cytokine environment of the prostate tissue restricts CXCR4-viral entry and/or spread. Thus, a high expression of the CXCR4 ligand CXCL12 (SDF-1) could impair X4 infection of prostate tissue, as previously described for the female genital mucosa [41,42]. However, the fact that viral DNA accumulation in prostate explants was similar for R5_SF162 _and X4_IIIB _up to 7 days post-infection indicated that both strains entered target cells and retrotranscribed their RNA genome following virus exposure of prostate explants. Afterwards, only R5 efficiently propagated in the explants, whilst a much more modest rise in DNA was observed for X4_IIIB _after 17 days. This suggests that the limited X4 spread in prostate tissue is not due to an entry inhibition but rather resulted from either a slower replication kinetic or a more restricted number of cells that supported efficient replication following entry. In cervical explants, a post-entry block due to the non-activated status of T lymphocytes was described for lab-adapted X4 and primary isolates of X4, R5X4, or R5. This block was compensated for in the prototypic R5_BaL _strain by its efficient spreading in macrophages, while infecting only a few T lymphocytes [37,43]. Although the prototypic R5_SF162 _strain used in our study can also efficiently spread in macrophages [44], the fact that the main infected cell type for both R5 and R5X4 was T lymphocytes, indicated that the difference between R5/R5X4 and X4 strains was not due to exclusive replication of the formers in macrophages, a cell type that X4 strains cannot productively infect [45]. One hypothesis is that the limited replication of X4_IIIB _is due to a sub-optimal level of activation of its target cells. Thus, sub-optimally stimulated CD4+T cells have been shown to only support the early steps of X4 viral replication. Whilst X4 replication in lymphocytes is a function of the time interval from mitogenic stimulation, R5 viruses require less stringent conditions of T-cell activation and are able to replicate in all subsets of sub-optimally activated T lymphocytes [46]. Since inflammatory infiltrates of BPH prostate tissues are mainly constituted of chronically activated CD4+ T cells, the activation status of those cells (i.e chronically rather than acutely activated) may allow efficient R5, but no X4, replication.

## Conclusion

Our results demonstrated that the prostate is a site of R5 virus replication through the infection of the immune cells present in the tissue. The less efficient spread of HIV-1 X4 virus observed in the prostate explants could contribute to the preferential sexual transmission of HIV-1 R5 strains. Whether restricted X4 replication occurs in the prostate of HIV patients awaits further investigations, since different conditions such as the level of immune cell activation and the cytokine environment may modify X4 virus replication in the prostate *in vivo*.

## Competing interests

The authors declare that they have no competing interests.

## Authors' contributions

ALT, APS, HD, NRL, LH performed the experiments; NRL, AR, BJ contributed reagents/materials/analysis tools; ALT, APS, HD, NLR, AR, ND analyzed the data; ND conceived and designed the experiments.
